# Thrombelastography (TEG^®^): practical considerations on its clinical use in trauma resuscitation

**DOI:** 10.1186/1757-7241-21-29

**Published:** 2013-04-16

**Authors:** Luis Teodoro da Luz, Bartolomeu Nascimento, Sandro Rizoli

**Affiliations:** 1 Department of Critical Care Medicine. Sunnybrook Health Science Centre, University of Toronto, Toronto, ON, Canada; 2 Department of Surgery, Sunnybrook Health Sciences Centre, University of Toronto, Toronto, ON, Canada; 3 Surgery and Critical Care Medicine, Departments of Surgery and Critical Care Medicine, St Michael’s Hospital, University of Toronto, M5B 1W8, Toronto, ON, Canada

**Keywords:** Thrombelastography, TEG^®^, Fibrinolysis, Trauma coagulopathy

## Abstract

**Background:**

Thrombelastography is a laboratorial test that measures viscoelastic changes of the entire clotting process. There is growing interest in its clinical use in trauma resuscitation, particularly for managing acute coagulopathy of trauma and assisting decision making concerning transfusion. This review focuses on the clinical use of thrombelastography in trauma, with practical points to consider on its use in civilian and military settings.

**Methods:**

A search in the literature using the terms “thrombelastography AND trauma” was performed in PUBMED database. We focused the review on the main clinical aspects of this viscoelastic method in diagnosing and treating patients with acute coagulopathy of trauma during initial resuscitation.

**Results:**

Thrombelastography is not a substitute for conventional laboratorial tests such as INR and aPTT but offers additional information and may guide blood transfusion. Thrombelastography can be used as a point of care test but requires multiple daily calibrations, should be performed by trained personnel and its technique requires standardization. While useful partial results may be available in minutes, the whole test may take as long as other conventional tests. The most important data provided by thrombelastography are clot strength and fibrinolysis. Clot strength measure can establish whether the bleeding is due to coagulopathy or not, and is the key information in thrombelastography-based transfusion algorithms. Thrombelastography is among the few tests that diagnose and quantify fibrinolysis and thus guide the use of anti-fibrinolytic drugs and blood products such as cryoprecipitate and fibrinogen concentrate. It may also diagnose platelet dysfunction and hypercoagulability and potentially prevent inappropriate transfusions of hemostatic blood products to non-coagulopathic patients.

**Conclusions:**

Thrombelastography has characteristics of an ideal coagulation test for use in early trauma resuscitation. It has limitations, but may prove useful as an additional test. Future studies should evaluate its potential to guide blood transfusion and the understanding of the mechanisms of trauma coagulopathy.

## Review

Two major and recent advances in trauma are the resuscitation of massive bleeding patients with reconstituted whole blood (transfusion of plasma, platelet and red blood cell at a 1:1:1 ratio) and an increasing understanding of the pathophysiology of the acute coagulopathies of trauma [1,2]. These advances have been accompanied by an unprecedented interest on the potential clinical applications of thrombelastography (TEG^®^), a viscoelastic laboratory method of hemostasis testing for both civilian and military trauma care. While the number and quality of the studies on TEG^®^ in trauma is gradually increasing, many of its potential applications remain unclear to practicing surgeons, who characteristically are unfamiliar with laboratory technologies. This review focuses on the potential practical clinical application of thrombelastography for both civilian and military trauma settings.

## Brief overview – test methodology

**Figure 1:**
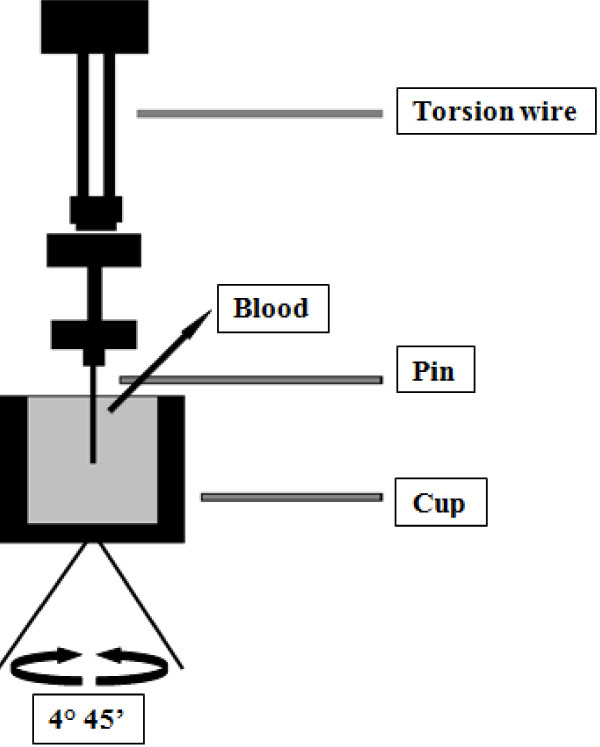
**The Principles of the Thromboelastography.** The viscoelastic properties of the clot are measured. Whole blood is inserted in the cup. A torsion wire suspends a pin immersed in the cup and connects with a mechanical–electrical transducer. The cup rotates through 4°45^′^ to imitate sluggish venous flow and activate coagulation. The speed and strength of clot formation is measured in various ways (now usually by computer), and depends on the activity of the plasmatic coagulation system, platelet function and fibrinolysis.

**Figure 2:**
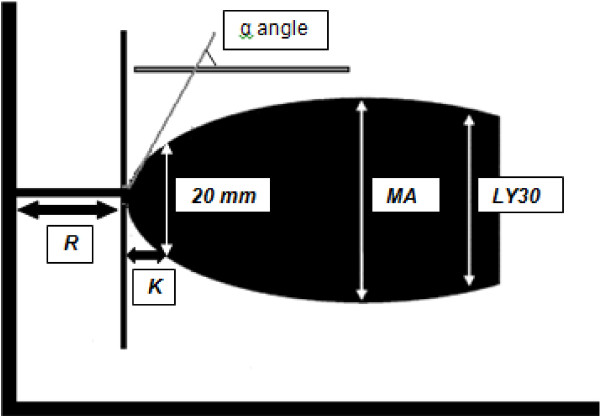
**A representative signature waveform of a normal TEG^®^ tracing.** R(seconds) = time of latency from start of test to initial fibrin formation; K (seconds) = time taken to achieve a certain level of clot strength (amplitude of 20mm); α angle (degrees) = measures the speed at which fibrin build up and cross linking takes place, hence assesses the rate of clot formation; MA (millimeters) = represents the ultimate strength of the fibrin clot; LY30 (%) = percentage decrease in amplitude at 30 minutes post-MA and gives measure of degree of fibrinolysis.

**Figure 3:**
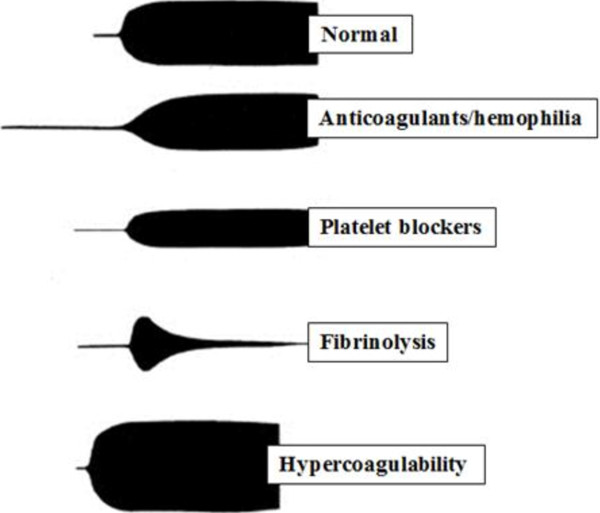
**Examples of normal and abnormal tracings on TEG^®^.** Normal (R, K, alpha and MA are normal); patient in use of anticoagulants or with hemophilia – deficit of coagulation factors (R, K are prolonged and alpha and MA are decreased); platelet dysfunction (R is normal, K is prolonged and MA is decreased); fibrinolysis (R is normal and MA is continuously decreases) and hypercoagulability (R, K are decreased and alpha and MA are increased).

Thrombelastography or TEG^®^ is based on the principle that the end result of the hemostatic process is a clot and its physical properties determine the patients’ hemostatic status. This test measures the physical properties of the clot in whole blood via a pin suspended in a cup from a torsion wire connected with a mechanical–electrical transducer (Figure 1). As the blood sample clots, the changes in rotation of the pin are converted into electrical signals that a computer uses to create graphical and numerical output [3]. A representative signature waveform is shown in Figure 2 while common abnormalities are presented in Figure 3. There are in fact two commercially available analogous systems, the thrombelastography (TEG^®^, Hemoscope Corporation, Niles, IL) and the rotational thromboelastometry (ROTEM®; Tem International GmbH, Munich, Germany). Both use the technique described by Hartert [4] with slightly different nomenclatures [5] and some technical differences. While differences in diagnostic nomenclature for identical parameters exist between the two systems, the primary hardware difference between the systems is that TEG^®^ operates by moving a cup in a limited arc (±4°45^′^ every 5s) filled with sample that engages a pin/wire transduction system as clot formation occurs, whereas the ROTEM® has an immobile cup wherein the pin/wire transduction system slowly oscillates (±4°45^′^ every 6s) [5]. Because the authors’ experience is with thrombelastography [3], this manuscript will focus on this system.

### Practical considerations about TEG^®^

#### Thrombelastography is not similar to conventional coagulation tests

TEG^®^ is a different test in that it measures the viscoelastic properties of whole blood as it clots. It provides global information on the dynamics of clot development, stabilization and dissolution which reflects *in vivo* hemostasis. It assesses both thrombosis and fibrinolysis [6,7]. Many studies have attempted to correlate TEG^®^ with standard coagulation tests and concluded that the association is weak. An important reason for this difference may reside in the fact that conventional tests are performed in plasma without platelets and tissue bearing cells (the cellular component) while TEG^®^ is done in whole blood. Jeger [8] reported the poor correlation between TEG^®^ and international normalized ratio (INR), activated partial thromboplastin time (aPTT) and platelet count. Among 31 combat casualties Doran [9] reported 64% of the patients on arrival had abnormal TEG^®^ values but only 10% of the same patients had abnormal conventional coagulation lab tests. On the other hand, Cotton [10] in a pilot study with 583 consecutive trauma activations evaluated the timeliness of r-TEG^®^ results, their correlation with conventional laboratory tests, and the ability of r-TEG^®^ to predict early blood transfusion. He was able to demonstrate that ACT, *r*-value, and *k*-time are strongly correlated with PT, INR and aPTT whereas and α-angle is correlated with platelet count. His cohort of patients showed that the graphical r-TEG^®^ results are available within minutes, correlate with conventional coagulation tests that are not as rapidly available, and are predictive of early transfusions of packed red blood cells, plasma, and platelets.

Standard coagulation tests such as INR, aPTT have limitations in diagnosing coagulopathy and directing its treatment in trauma, and predict bleeding and the need for blood transfusion poorly [11]. In contrast TEG^®^ has been successfully used to guide blood and blood products transfusion both in cardiac surgery and liver transplantation [12-15]. In these areas, TEG^®^-based transfusion algorithms resulted in reduced blood usage [16]. There is some evidence that for the similar system ROTEM®, the measurements may closer resemble standard laboratory tests than TEG^®^ as described by Rugeri [16] that showed a strong association between PT, aPTT, fibrinogen and platelets with ROTEM® INTEM (assessment of clot formation, fibrin polymerization and fibrinolysis via the intrinsic pathway), EXTEM (assessment of clot formation, fibrin polymerization and fibrinolysis via the extrinsic pathway) and FIBTEM (ROTEM® analysis without platelets: qualitative assessment of fibrinogen status).

#### TEG^®^ can be a point of care (POC) test

Most conventional coagulation laboratory tests including PT, INR, aPTT, thrombin time (TT), platelet function and D-dimer can be done near the patient as a POC test [17]. Most hospitals however, are set up to have these tests done in a laboratory and because POC tests have limitations, their use as POC has been restricted. There are however, considerable advantages to have POC tests during trauma resuscitation since many decisions cannot wait for the usually long turnaround time of conventional lab tests. The management of massive bleeding in trauma requires prompt availability of lab test results. POC is clearly faster. In particular when A10 (amplitude at 10 minutes) is used, which has been shown useful to early estimate the entire clot firmness [18-20] if they are to be useful at all. Frequently, laboratories are unable to respond to this time demand and most critical decisions in massively bleeding trauma patients are taken without them. Since the use of TEG^®^ is being pushed forward for use in early trauma resuscitation, it is likely that many institutions will prefer to use it as POC rather than a hospital laboratory test.

In fact, many studies have used TEG^®^ in trauma as a POC test [17,21-25]. However its proper use as POC raises some considerations as follows:

##### TEG^®^ requires daily calibration

This system has been criticized for not undergoing the same evaluation process as conventional tests, and the lack of quality assessment program. In practice electronic quality assurance should be run daily or with each set of tests using commercial lyophilized samples [17]. The manufacturer recommendation is to calibrate the equipment 2 to 3 times a day. So any consideration of having the TEG^®^ equipment in the trauma room as POC requires a quality assurance program in place that includes multiple daily calibrations.

##### TEG^®^ as POC should not be done by untrained personnel

It is imperative that some form of training and qualification, besides quality assurance methodology, must be put in place for anyone performing the test, as required by regulatory agencies such as American Association Blood Banks, Clinical Laboratory Improvement Amendments (CLIA) and Food and Drug Administration (FDA).

##### TEG^®^ as POC requires standard techniques

A major limitation of TEG^®^ is the many technical variations in how it is done, and the different results each technique generates [3]. TEG^®^ was originally designed for fresh whole blood and no additional activator [17]. Subsequent modifications have included sample anticoagulation and use of an activator to standardize the initiation of the coagulation [17,26].

In native whole blood the test should be done within 4 minutes while for recalcified citrated whole blood the recommendation is to wait 30 to 40 minutes for maximum stability, before doing the test [3]. This recommendation is sometimes neglected in trauma. The results from citrated blood are not comparable to native blood, thus anticoagulated samples are surrogate to native blood in assessing coagulation [26]. Furthermore each activator has indications with Kaolin being best for overall assessment of the coagulation; ADP and arachdonic acid for platelet function and tissue factor for evaluation of the extrinsic pathway and rapid TEG^®^[21]. Other studies showed that repeated sampling results in false hypercoagulable results [26] while gender, age and even alcohol also affect the TEG^®^ results [3,12,27].

##### TEG^®^ may not be faster than standard tests

While meaningful information can be obtained in approximately 10 minutes of initiating the test, when maximum clot firmness is reached [9], the whole test takes 30 to 60 minutes [17]. This time may be increased by the recommended 30 to 40-minute wait for anticoagulated samples [17]. Rapid TEG^®^ (rTEG^®^) was recently proposed where the test is accelerated by a solution containing tissue factor (TF) [28]. Kashuk [29] proposed that rTEG^®^ is best done in non-citrated whole fresh blood samples within 4 minutes of sampling. According to this study, rTEG^®^ is superior to conventional lab tests to guide blood transfusion and its tracing parallel the 3 steps of coagulation: initiation, amplification and propagation.

A study by Jeger [8] reported that rTEG^®^ took 30.8 ± 5.7 minutes, Kaolin-initiated TEG^®^ 41.5 ± 5.6 while conventional lab tests 64.9 ± 18.8 minutes from admission to results being available. These figures are not impressive compared to time from sampling to results (reported by telephone directly to the trauma team leader) in our institution of 40 minutes for the INR [30]. It also is not impressive compared to a recent publication by Chandler [31] of an emergency lab panel of PT, fibrinogen, platelet count and hematocrit to help the management of massive bleedings, which is available in 14 ± 3 minutes. A more recent publication by Cotton [10] showed that the early r-TEG^®^ values (ACT, *k*-time, and *r*-value) were available within 5 minutes, late r-TEG^®^ values (MA and α-angle) within 15 minutes, and PBCTs within 48 minutes (*p* < 0.001).

#### TEG^®^ measures clot strength

Possibly the most important information provided by TEG^®^ is clot strength. It may help identify whether the source of bleeding is a failure of clot formation (coagulopathy) or a mechanical bleeding [21]. Clot strength is also the key information in guiding transfusion of blood products, and has a central role in any TEG^®^-based transfusion algorithm. Clot strength is measured by the maximal amplitude (MA -represents the ultimate strength of the fibrin clot), which is affected by fibrinogen, platelet (number and function) and factor XIII. Bowbrick [32] demonstrated a strong linear correlation between log platelet count and MA. Maximal amplitude is the greatest amplitude of the tracing and reflects platelet contribution to clot strength. Low MA values correspond with states of platelet dysfunction or hypofibrinogenemia [10]. Another measurement of clot strength as the end result of the interaction of platelets and fibrin is the shear elastic modulus strength or G. G is a computer generated value reflecting the strength of the clot from the initial fibrin burst through fibrinolysis G = (5000 × amplitude)/ (100 – amplitude) normal = 5.3–12.4 dynes/cm2. According to Kashuk [7], G is the best measurement of clot strength. The trauma group from Denver proposed a TEG^®^–based transfusion algorithm where G value is the first step and MA is a key element [29]. While there are not reports on the use of this algorithm or any validation published, the Denver group proposes that TEG^®^-based algorithm is superior to conventional lab tests such as INR, fibrinogen, platelet count to guide transfusion. In this same manuscript, they also report that r-TEG^®^ had been used instead of INR to guide transfusion, that less frozen plasma would have been given to trauma patients. On platelet or cryoprecipitate transfusion, rTEG^®^ would have had no impact on the amount transfused. The same group from Denver recently published a study addressing the functional fibrinogen test [33] that can be performed rapidly with TEG^®^ and correlates well with the standard fibrinogen assay. Both fibrinogen and platelet contribution of clot strength can be derived from functional fibrinogen. Moreover, functional fibrinogen had a stronger correlation to clot strength, and increased levels were directly associated with increased percent contribution to clot strength. In vitro studies also demonstrated an increase in functional fibrinogen, clot strength, and percent fibrinogen contribution to clot strength with the addition of fibrinogen concentrate. These data suggest that fibrinogen should be addressed early in trauma patients manifesting acute coagulopathy of trauma. Plotkin [23], in a retrospective study of 44 patients with penetrating injuries from a combat hospital, demonstrated that, despite the INR and aPTT being abnormal in 39% and 37% of the patients respectively, they had no association with transfusion. In contrast, abnormal TEG^®^ in the first 24 hours of admission, including abnormal MA, was significantly associated with blood transfusion and low platelet count. Nystrup [34] more recently showed that clot strength correlated with the amount of packed red blood cells (*p* = 0.01), fresh frozen plasma (*p* = 0.04) and platelet concentrates (*p* = 0.03) transfused during the first 24 hours of admission and in the same study, patients with low clot strength demonstrated increased 30-day mortality (47% vs. 10%, *p* < 0.001). Pezold [35] showed that G was independently associated with massive transfusion and death. Kashuk [36] have concluded that the reduction in clot strength is indicative of transfusion requirement in penetrating injuries and propose MA to guide transfusion. MA seems to correlate with fibrinogen, platelets and activated FXIII, so diagnoses of these coagulation impairments may be possible with TEG^®^^.^

#### TEG^®^ measures fibrinolysis

The role of fibrinolysis in early trauma coagulopathy is poorly understood but growing evidence suggests it is a major player, at least for some patients [21,23-25]. The best evidence on the importance of excessive fibrinolysis in trauma comes from the CRASH-2 study. This large randomized control trial showed that trauma patients at risk for, or with ongoing bleeding receiving an anti-fibrinolytic agent within 3 hours of the trauma have a significant reduction in mortality. Thus early diagnosis of hyperfibrinolysis could not only guide the use of anti-fibrinolytic drugs but also the administration of fibrinogen concentrate or cryoprecipitate, while ruling it out could prevent their inappropriate use. Fibrinolysis however, is a difficult laboratorial diagnosis that requires multiple tests such as platelet count, coagulation factors, fibrinogen, protein S, C and antithrombin [37]. Most of these tests are not available for use in early trauma resuscitation while others such as D-dimer, a marker of fibrinolysis, might not be useful since it is elevated in virtually all trauma patients [38]. TEG^®^ and ROTEM® are the only tests available that can rapidly diagnose and quantify fibrinolysis during early trauma resuscitation.

Fibrinolysis is measured by clot lysis index as per Kang [39] or estimated percent lysis (EPL), which are mathematical derivations from the amplitude [40,41]. Kashuk [7] arbitrarily defined primary fibrinolysis as EPL >15 and coagulopathy as clot strength G <5.3 dynes/cm^2^ and reported that among 61 patients, 11 (18%) had primary fibrinolysis on arrival, which corresponded to 34% of those requiring massive transfusion. Patients with primary fibrinolysis were significantly more hypotensive, hypothermic and received more transfusions. They also had lower fibrinogen levels and higher mortality. An interesting observation is that fibrinolysis was a different condition from the hypocoagulable state. Mortality was also referred in the study of Theusinger [42] where in the trauma hyperfibrinolytic group the mortality was 77% ± 12%, significantly higher than in the nontrauma hyperfibrinolytic group (41% ± 10%; *p <* 0.001, 95% CI 5%–67%). In a very recent study by Cotton [43] hyperfibrinolysis was significantly (*p* = 0.017) associated with mortality in trauma patients. Ives [44], in a recent study showed also that hyperfibrinolysis was a strong predictor of early (24 hours) mortality (odds ratio = 25.0; 95% CI, 2.8–221.4; *p* = 0.004), predicting 53% of early deaths.

Other studies on fibrinolysis in trauma were made with ROTEM®. Rugeri [45] demonstrated that 25 out of 90 trauma patients (28%) had evidence of abnormal fibrinolysis. A more recent study by Schöchl [25] on 33 patients over a 4 year period described 3 patterns: fulminant (within 30min), intermediate (30–60 min), late >60 min. For those with fulminant hyperfibrinolysis the mortality was 100%. The authors proposed that ROTEM® is the gold standard to diagnose hyperfibrinolysis, being superior to other laboratory tests. Levrat [24] compared ROTEM® to gold standard lab tests such as euglobulin lysis time (ELT), D-dimer and fibrinogen level. In this study hyperfibrinolysis was defined as ELT < 90 min or ROTEM® clot amplitude, maximum clot firmness (MCF), clot lysis index plus the ability of aprotinin to reverse these parameters. Among 23 trauma patients, 6% had hyperfibrinolysis by ELT, MCF (that corresponds to MA in TEG^®^) and clot amplitude at 15 min, which had the best association [21]. As expected patients with hyperfibrinolysis had higher ISS (injury severity score), lower hemoglobin, platelet count, higher D-dimer and higher mortality.

#### TEG measures hypercoagulability and platelet dysfunction

It has been proposed that short R (time of latency from start of test to initial fibrin formation) and accelerated clot propagation on TEG^®^ indicates hypercoagulability, and as such TEG^®^ is an excellent test to diagnose this condition [2]. The clinical implication of such diagnosis is unknown since it may be a physiologic response to trauma and blood loss, but may also be associated with thromboembolic complications. Tuman [46] in a study with severe bleeding trauma patients, showed that only less than half of the patients bleeding near the entire blood volume become hypocoagulable (coagulopathic), demonstrating that bleeding and coagulopathy are different entities and thus indiscriminate transfusion of plasma and platelets to all bleeding patients may be inappropriate. Watters [47] identified evidence of hypercoagulability following splenectomy for trauma and an association with thromboembolic events and infection. Cotton [48], in a study with trauma patients who developed pulmonary embolism, demonstrated that these patients had an elevated MA and a higher ISS. MA was identified as predictor of pulmonary embolism, after controlling for gender, race, age and ISS. Carroll [49] and Nekludow [50] have demonstrated in trauma patients and Vucelic [41] in elective surgery that TEG^®^ may diagnose platelet dysfunction. Platelet dysfunction is believed to have an important role in traumatic bleeding. The combined effects of shock tissue destruction and hypothermia could affect platelet function via mechanisms that have been poorly investigated in trauma [51]. TEG^®^ (platelet mapping) and also ROTEM® have developed specific assays to evaluate platelet function, which are mainly used in elective surgery [17]. The utility of these assays have not yet been fully examined and their usefulness not determined.

### TEG^®^-Based transfusion algorithm for trauma

Thrombelastography has been criticized for not being subjected to the same evaluation processes as many conventional lab tests. It has however, been shown to reduce the use of blood products in both cardiac surgery [52,53] and liver transplantation [15], thus it is plausible that it can do the same for trauma. There are currently 2 options for the management of massive bleedings in trauma. Damage control resuscitation proposes empirical resuscitation with aggressive transfusion of red blood cells, fresh plasma and platelets. The “classical” resuscitation proposes transfusion based on conventional laboratory tests. Possibly most massively bleeding trauma patients are resuscitated nowadays by a combination of the two options. The possibility that laboratorial tests such as TEG^®^ could guide blood transfusion is attractive and a few TEG^®^-based transfusion algorithms for trauma have recently been proposed [2,6,35,36,54,55]. However, the processes used to create these algorithms are missing, and no prospective validation or comparisons to other algorithms have been reported. The additional difficulty in using the proposed algorithms is that the threshold TEG^®^ values that justify transfusion are unknown. In a similar way that a hemoglobin level of 105 g/L despite being abnormal would not support blood transfusion in most instances; it becomes difficult to justify transfusing platelets or plasma simply because the MA value is 53 when the normal is ≥54 as in the algorithms. Another criticism to TEG^®^-based algorithms is its mono-analysis with a single activator (Kaolin) compared to algorithms based on multiple assays (i.e. ROTEM®) with improved diagnostic efficacy [56]. Harr [33] recently pointed out that TEG inability to differentiate between fibrinogen and platelet contribution to clot firmness resulted in two TEG^®^-based algorithms proposing different interventions to treat the same problem (low clot strength).

## Conclusions

Hemostatic interventions such as blood transfusions may be life saving for a coagulopathic trauma patient. In contrast, for a patient with mechanical source of bleeding and hypercoagulable, the same interventions may delay bleeding control and increase both morbidity and mortality. TEG^®^ offers the possibility of quickly identifying whether the bleeding is due to incompetent clotting (coagulopathy), excessive clot lysis (hyperfibrinolysis), both or neither one. This information can then be used to guide appropriate use of blood, blood products and other interventions such as antifibrinolytics. It may also be used to prevent inappropriate treatments.

TEG^®^ has many of the characteristics of an ideal coagulation test for trauma resuscitation [5]. It however, is one more option for diagnosis and requires further work particularly regarding standardization before its potential can be fully evaluated across different institutions [3]. Future studies should focus on the use of TEG^®^ in guiding blood transfusion as well as advancing the knowledge of the mechanisms of early trauma coagulopathies.

## Competing interests

Dr. Rizoli held a Canadian Institute of Health Research (CIHR) New Investigator award in partnership with NovoNordisk Canada, manufacturer of recombinant factor VIIa and is a member of the CSL Behring Scientific Advisory Board.

## Authors’ contributions

LTL, BN and SR were instrumental in the conception and design of this review, in acquiring data, analysing and interpreting it. All 3 authors were involved in drafting the manuscript and revising it critically. All 3 authors have given their final approval for the version to be published.
